# Peripartum cardiomyopathy

**DOI:** 10.1007/s00059-018-4709-z

**Published:** 2018-05-16

**Authors:** T. Koenig, D. Hilfiker-Kleiner, J. Bauersachs

**Affiliations:** Department of Cardiology and Angiology, Hannover Medical School, Carl-Neuberg-Str. 1, 30625 Hannover, Germany

**Keywords:** Cardiomyopathies, Prolactin, Heart failure, Pregnancy complications, Bromocriptine, Kardiomyopathien, Prolaktin, Herzinsuffizienz, Schwangerschaftskomplikationen, Bromocriptin

## Abstract

Peripartum cardiomyopathy (PPCM) is a rare and potentially life-threatening disease that occurs toward the end of pregnancy or in the months following delivery in previously heart-healthy women. The incidence varies widely depending on geographical region and ethnic background, with an estimated number of 1 in 1000–1500 pregnancies in Germany. The course of the disease ranges from mild forms with minor symptoms to severe forms with acute heart failure and cardiogenic shock. The understanding of the etiology of PPCM has evolved in recent years. An oxidative stress-mediated cleaved 16-kDa fragment of the nursing hormone prolactin is thought to damage endothelial cells and cardiomyocytes. Bromocriptine, a dopamine-receptor agonist, effectively blocks prolactin release from the pituitary gland. In addition to standard heart failure therapy, this disease-specific treatment reduces morbidity and mortality in PPCM patients. This review summarizes the current knowledge on PPCM and the disease-specific treatment options.

## Zusammenfassung

Die Peripartum-Kardiomyopathie (PPCM) ist eine seltene und potenziell lebensbedrohliche Erkrankung, die gegen Ende der Schwangerschaft oder in den Folgemonaten nach Entbindung bei zuvor herzgesunden Frauen auftritt. Die Inzidenz der PPCM variiert mit deutlichen regionalen und ethnischen Unterschieden. In Deutschland beträgt die geschätzte Inzidenz etwa 1 von 1000–1500 Schwangerschaften. Der klinische Verlauf umfasst leichte Formen mit unspezifischen Symptomen bis hin zu einer schweren akuten Herzinsuffizienz mit Entwicklung eines kardiogenen Schocks. In den vergangenen Jahren ist das Stillhormon Prolaktin in den Fokus der Pathogenese der PPCM gerückt. Durch vermehrten oxidativen Stress wird eine Spaltung des Prolaktins in ein toxisches 16-kDa-Fragment induziert. Dieses schädigt sowohl Endothelzellen als auch Kardiomyozyten und resultiert in einer systolischen Herzinsuffizienz. Bromocriptin, ein Dopaminrezeptoragonist, hemmt die Freisetzung des Prolaktins aus der Hypophyse. Durch die Bromocriptinbehandlung in Kombination mit einer medikamentösen Herzinsuffizienztherapie lassen sich die Morbidität und Mortalität der PPCM senken. In der vorliegenden Übersichtsarbeit werden die aktuellen wissenschaftlichen sowie klinischen Erkenntnisse zur PPCM zusammengefasst und die spezifischen Therapieoptionen erläutert.

The global burden of pregnancy-associated heart failure contributes substantially to maternal morbidity and mortality [1, 2]. Hemodynamic alterations that affect the maternal circulatory system primarily occur during late pregnancy and the delivery phase [3]. Therefore, women with either congenital or acquired cardiomyopathies are particularly affected by these changes as heart function can be altered or even worsen [4]. Approximately 4% of postpartum hospitalizations are due to maternal myocardial disorders with increasing tendency [5]. Against the background of rare evidence-based treatment options, physicians are often faced with diagnostic and therapeutic challenges. Peripartum cardiomyopathy (PPCM) represents a life-threatening, yet underdiagnosed pregnancy-associated heart disease seriously affecting young women [6–8]. This review summarizes the current knowledge on PPCM and presents disease-specific treatment strategies.

## Definition, epidemiology, and risk factors

PPCM is defined as an idiopathic cardiomyopathy with systolic heart failure occurring toward the end of pregnancy or in the month following delivery in previously healthy women. As defined by the Study Group on PPCM of the Heart Failure Association (HFA) of the European Society of Cardiology (ESC), the left ventricular ejection fraction (LVEF) is nearly always <45% [9]. PPCM is a diagnosis of exclusion. Other causes of heart failure (e. g., pregnancy-associated myocardial infarction or pulmonary embolism) and pre-existing heart disease (e. g., congenital heart disease or chemotherapy-induced toxic cardiomyopathy) should be ruled out by thorough history-taking and extensive clinical work-up [10]. Although resembling the characteristics of dilated cardiomyopathy, PPCM is considered an independent entity [11, 12].

The global incidence of PPCM varies widely with considerable regional and ethnic differences. Several regions have been described as hot spots, such as Nigeria and Haiti with an incidence of 1 in 100 and 1 in 300 pregnancies, respectively [9]. The estimated incidence of PPCM in Germany is approximately 1:1000–1500 and is comparable to numbers from South Africa (1:1000) and the United States (1:1500) [10, 13, 14]. It should be emphasized that especially milder forms of PPCM may be undiagnosed owing to unspecific symptoms and reduced awareness. Mortality rates range from 2 to 30% when analyzing worldwide data [9]. However, recent German data demonstrated an even lower mortality rate (0%) in PPCM patients receiving the prolactin blocker bromocriptine on top of standard heart failure therapy [15].

Several risk factors have been identified in recent years. Major risk factors for developing PPCM are pregnancy-associated hypertensive disorders such as gestational hypertension, preeclampsia, or HELLP syndrome (hemolysis, elevated liver enzymes, low platelets) [16]. Older maternal age, multifetal pregnancies, multiparity, and in vitro fertilization have also been suggested to increase the risk of PPCM [8, 17]. Moreover, women with an African ancestry have a higher risk than Caucasian and Asian women [18–20].

## Pathophysiology of PPCM

The pathophysiology of PPCM is still largely unknown, although several mechanisms have been proposed, including autoimmune processes, inflammatory factors, viral infections, and low selenium levels [6, 21]. However, none of these mechanisms has been fully proven yet. In recent years, a shift in the angiogenic balance toward an anti-angiogenic environment has emerged as a potential initiating and driving factor of PPCM [22, 23]. Among these upregulated anti-angiogenic factors, the cleaved N‑terminal 16-kDa prolactin fragment and sFlt-1 are considered crucial [23, 24].

### Prolactin pathway

The milestone of current disease-specific PPCM treatment strategy was introduced in 2007. Hilfiker-Kleiner and colleagues demonstrated the pivotal role of the nursing hormone prolactin in a mouse model with a cardiomyocyte-specific knockout of the signal transducer and activator of transcription factor-3 (STAT3) [24]. Due to increased oxidative stress, the full-length 23-kDa prolactin is cleaved into the anti-angiogenic, pro-inflammatory, and pro-apoptotic 16-kDa prolactin fragment by proteolytic enzymes such as cathepsin D and matrix metalloproteinases. The 16-kDa fragment, also called vasoinhibin, directly impairs endothelial function and triggers the release of micro-RNA 146a, which in turn has detrimental effects on cardiomyocytes [25]. This ultimately results in systolic heart failure that is potentially reversible. The diseased myocardium was salvaged by specific treatment with bromocriptine that blocks prolactin release from the pituitary gland and, therefore, prevents the cleavage of the full-length prolactin into the toxic 16-kDa fragment [6, 24].

### sFlt-1 pathway

A soluble receptor of the vascular endothelial growth factor (VEGF), the so-called sFlt-1 (soluble fms-like tyrosine kinase-1), plays an important role in the pathophysiology of preeclampsia [26]. sFlt-1 is produced, inter alia, in the placenta during late pregnancy and is associated with a systemic angiogenic imbalance [22, 23]. Patten and colleagues demonstrated that vascular dysfunction caused by upregulated sFlt-1 also induces PPCM with severe impairment of cardiac function in a mouse model lacking cardiac peroxisome proliferator-activated receptor gamma coactivator 1‑alpha (PGC-1α) [23]. However, a pro-angiogenic therapy with VEGF alone had no beneficial effect in mice. The combination of bromocriptine and recombinant VEGF ultimately rescued these mice. These results further strengthen the importance of prolactin but also of other anti-angiogenic factors. Whether an additional treatment of human PPCM patients with a recombinant VEGF has beneficial effects has remained unclear to date.

## Genetics

Recent data suggest that about 15% of PPCM patients display mutations in genes associated with dilated cardiomyopathy strengthening the concept of a shared genetic background [11]. Several affected genes have been previously described, such as cardiac myosin heavy chain (MYH), titin (TTN), and SCN5 [11, 27]. Most of the mutation carriers are asymptomatic prior to pregnancy. During late pregnancy, delivery, and in the early postpartum period, women are faced with profound hemodynamic changes (increase in heart rate and cardiac stroke volume, decline in total peripheral vascular resistance and volume overload; [1, 4]) and it seems that this hemodynamic stress may unmask genetic cardiomyopathies.

## Signs and symptoms

The course of the disease ranges widely from milder forms with only slight and unspecific symptoms to severe forms with life-threatening cardiogenic shock [7, 10, 19]. Physicians are often faced with supposedly healthy women toward the end of their pregnancy or after delivery who complain of unspecific symptoms such as general discomfort, fatigue, and peripheral edema. These symptoms often mimic common complaints in the peripartum period. By contrast, more severely affected women suffer from dyspnea, orthopnea, and agitation. At worst, cardiogenic shock with pulmonary edema and peripheral hypoperfusion can be observed [7].

## Diagnostics

**Fig. 1:**
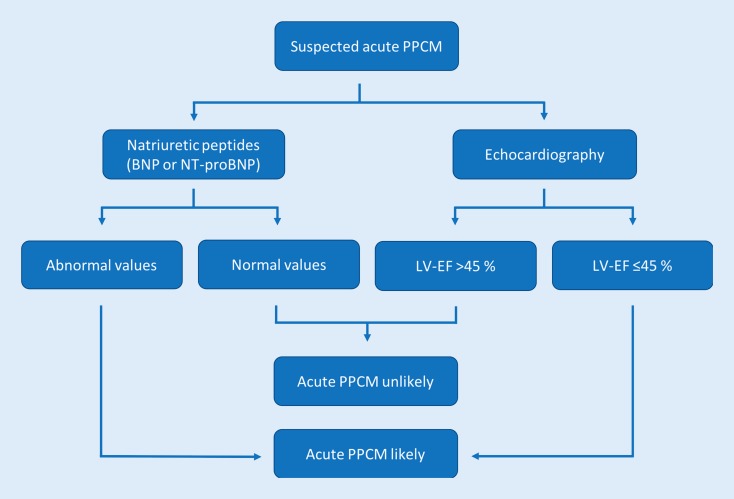
Pragmatic evaluation scheme for suspected acute PPCM during the end of pregnancy or the months after delivery. Measurement of natriuretic peptides and transthoracic echocardiography are recommended to quickly strengthen or rule out the diagnosis of PPCM. *PPCM* peripartum cardiomyopathy, *LVEF* left ventricular ejection fraction, *BNP* brain natriuretic peptide, *NT-proBNP* N-terminal prohormone brain natriuretic peptide

The importance of an early diagnosis has to be emphasized [28]. Therefore, the initiation of appropriate treatment should not be delayed by the diagnostic work-up. Physical examination mainly reveals signs of congestion such as pulmonary rales, peripheral edema, and/or jugular vein distension. Women can be pale and, in cases of cardiogenic shock, present with cold and wet skin due to centralization and volume overload [7]. In instances where pregnancy-associated heart disease is suspected, two crucial diagnostic tests should be immediately performed (Fig. 1): Measurement of circulating natriuretic peptides is recommended in every patient with suspected heart failure. Currently, either brain natriuretic peptide (BNP) or its N‑terminal prohormone (NT-proBNP) are screening biomarkers for heart failure [8, 29]. Although elevated levels are not specific for pregnancy-associated heart diseases, normal values can rapidly rule out acute heart failure. Furthermore, transthoracic echocardiography is widely available and can be easily performed at bedside. Left ventricular (LV) function can be quickly determined and, thus, the diagnosis of PPCM can be strengthened or excluded. Concomitant pathologies such as right ventricular involvement and mitral regurgitation can also be detected.

Cardiac magnetic resonance imaging (MRI) is not routinely performed because specific MRI features of PPCM are missing. However, the pivotal role of cardiac MRI in terms of characterization of myocardial tissue and assessing biventricular systolic function remains unique. Furthermore, differential diagnoses such as myocarditis or ischemic myocardial scars can be made. A coronary angiography is usually not necessary unless there are signs and/or symptoms of myocardial ischemia [7]. Similarly, an endomyocardial biopsy is not routinely recommended since therapeutic consequences are rare [10]. The electrocardiogram (ECG) is not specific for PPCM but allows for the detection of signs of myocardial ischemia/infarction.

## Therapy

The treatment of PPCM patients is primarily based on the guidelines for the diagnosis and treatment of acute and chronic heart failure published by the European Society of Cardiology [29], a practical guidance paper of the Study Group on PPCM of the Heart Failure Association of the European Society of Cardiology [7], and a landmark paper on the current management of PPCM [10]. Primarily, assessment of the patient’s hemodynamic status and evaluation of signs and symptoms of congestion and cardiogenic shock are crucial.

### Treatment of hemodynamically unstable acute PPCM

Women with acute heart failure and cardiopulmonary distress (e. g., systolic blood pressure <90 mm Hg, peripheral oxygen saturation <90%, or lactate >2.0 mmol/l) should be immediately transferred to the intensive care unit of an experienced center. Depending on the volume status, preload has to be optimized by either fluid administration or diuretics. If the systolic blood pressure is >110 mm Hg, vasodilators are recommended to improve afterload (during pregnancy preferably hydralazine). In cases of hemodynamic instability, inotropes and/or vasopressors may be necessary. However, it should be emphasized that catecholamines (especially dobutamine) potentially harm PPCM patients and therefore should be avoided whenever possible [30]. The administration of levosimendan, an inodilator and calcium sensitizer, appears to be safe and may be preferred [31]. Early evaluation of mechanical circulatory support (MCS) is recommended. Percutaneous devices (such as the Impella® [Abiomed, Danvers, MA, USA] microaxial pump) are favored in patients with isolated LV failure, while the combination of a percutaneous device and a veno-arterial extracorporeal membrane oxygenation (ECMO) is recommended in patients with biventricular failure. Additionally, termination of pregnancy by urgent delivery via caesarean section is mandatory in these cases. Corticosteroids for fetal lung maturation should be given in women up to the 34th week of gestation.

### Treatment of hemodynamically stable acute PPCM

In PPCM patients who present with hemodynamic stability without cardiopulmonary distress and with an ongoing pregnancy, therapy should focus on optimizing maternal hemodynamics and careful observation of the fetus. Heart failure therapy is restricted to beta-blockers, vasodilators (preferably hydralazine), and diuretics in cases of fluid overload [4]. Fetal lung maturity should be induced before week 34 of gestation. Vaginal delivery is the preferred mode of delivery in hemodynamically stable patients [4].

After delivery (regardless of whether the diagnosis of PPCM was made before or after delivery), standard heart failure therapy should be introduced [29]. Dedicated treatment regimens are described in detail elsewhere [7, 10, 29]. In summary, all women should be treated with an angiotensin-converting enzyme (ACE) inhibitor/angiotensin receptor blocker (ARB), and a beta-blocker in guideline-directed standard or maximally tolerated dosages. Mineralocorticoid receptor antagonists (MRA) are recommended in patients with an LVEF of <40%. Eplerenone should be preferred in young female heart failure patients because of fewer hormonal side effects and less blood pressure reduction. The angiotensin receptor neprilysin inhibitor valsartan/sacubitril should replace ACE inhibitors/ARBs in patients with persistent heart failure symptoms despite optimal medical heart failure therapy including ACE inhibitor/ARB, a beta-blocker, and an MRA. Ivabradine, an I_f_ channel inhibitor, should be considered in patients with sinus rhythm and inadequately controlled heart rates (>70 bpm at rest) even if beta-blockers are at maximally tolerated dosages [32]. Diuretics should be restricted to patients with fluid overload.

### Bromocriptine

Bromocriptine combines three benefits for PPCM patients: Firstly, stopping lactation is required to avoid the high metabolic demands of lactation and breastfeeding. Secondly, since most heart failure drugs are contraindicated during breastfeeding, ablactation is recommended to fully and safely introduce standard oral heart failure medication. Thirdly, inhibition of prolactin release by bromocriptine is used as a disease-specific treatment.

Based on encouraging experimental data and promising first clinical proof-of-concept studies, bromocriptine has been widely introduced into clinical practice [8, 33]. The results of a randomized multicenter German study comparing two bromocriptine regimens in PPCM patients were recently published [15]. A placebo group was not allowed in this trial by the ethics committees given the markedly positive effects of bromocriptine in smaller previous studies [8, 33]. In total, 63 women with an LVEF <35% were finally randomized to either a short-term (bromocriptine 2.5 mg once daily for 7 days) or a long-term regimen (bromocriptine 2.5 mg twice daily for 14 days followed by 2.5 mg once daily for another 42 days). All women obtained at least prophylactic anticoagulation during the entire bromocriptine therapy to avoid thrombotic complications. The primary endpoint was the change in LVEF assessed by cardiac MRI. In summary, the study was neutral in terms of LVEF change but there was a trend toward a better outcome in favor of the long-term group (delta LVEF 21% vs. 24% in the short- and the long-term regimen, respectively). This difference was even more pronounced when severely affected PPCM patients (LVEF <30% at study entry) were analyzed (delta LVEF 24% vs. 29% in the short- and the long-term regimen, respectively). Remarkably, there were no deaths and no need for LVAD implantation or heart transplantation. Bromocriptine treatment was safe with no serious adverse events. The results of a subgroup analysis (LVEF <30% at randomization) were compared with a cohort taken from the IPAC (Investigation or Pregnancy-Associated Cardiomyopathy) study [14]. In the United States, only a minority of PPCM patients (<1%) are treated with bromocriptine. Therefore, this cohort serves as a “control group”. Despite several limitations and potential confounding factors, the benefit of bromocriptine administration in PPCM patients was demonstrated. Whereas 37% of all patients enrolled in the IPAC study had a major event (left ventricular assist device implantation, heart transplantation, death) or an LVEF <35% at follow-up, there was only 1 of 37 (2.7%) patients who did not improve (LVEF <35%) during follow-up in the bromocriptine trial. In conclusion, bromocriptine treatment is safe and beneficial in PPCM patients. Since prevention of thromboembolic events is crucial, at least prophylactic anticoagulation is recommended during bromocriptine treatment.

### BOARD scheme

The BOARD scheme for the treatment of acute PPCM has been introduced recently [34]. This concept summarizes the currently recommended treatment in women after delivery. All patients should be treated with *B*romocriptine. The bromocriptine treatment scheme for PPCM patients of the Hannover Medical School is depicted elsewhere [15, 35]. *O*ral heart failure medication is recommended in standard or maximally tolerated dosages. Bromocriptine treatment should always be accompanied by at least prophylactic *A*nticoagulation to prevent thrombotic/thromboembolic events. Vaso*R*elaxing agents should be administered if systolic blood pressure is above 110 mm Hg to reduce afterload. *D*iuretics are recommended in cases of fluid overload.

### Prevention of sudden cardiac death

Although particular mechanisms remain unknown, sudden cardiac death (SCD) due to ventricular arrhythmias (ventricular fibrillation and sustained ventricular tachycardia) is not uncommon in patients with an LVEF ≤35% [36]. In a German retrospective analysis, a remarkably high number of six out of 49 PPCM patients (12%) suffered from life-threatening arrhythmia [37]. Most of the PPCM patients fully recover or at least improve significantly within 3–6 months and the risk of malignant arrhythmia thereby decreases. Hence, immediate implantation of a permanent cardioverter/defibrillator (ICD) does not appear to be appropriate. Instead, the use of a wearable cardioverter/defibrillator (WCD; LifeVest®, Zoll, Pittsburgh, PA, USA) is recommended to prevent SCD [7, 29, 37]. In patients who do not recover LV function (LVEF ≤35%) despite optimal medical therapy or those who experience ventricular tachycardia with hemodynamic instability or ventricular fibrillation, implantation of an ICD (either transvenous or subcutaneous) or a cardiac resynchronization device defibrillator (CRT-D) is recommended according to current guidelines [29].

## Prognosis

Although the clinical course can vary immensely, the overall prognosis of PPCM patients when treated according to current guidelines and recommendations is favorable. Approximately 50% of women fully recover (defined as LVEF >55% and NYHA class I), whereas another 35–40% partially recover (defined as improvement of LVEF >10% and at least one NYHA class) [8]. Fortunately, only a minority of women remain in NYHA class III/IV with persistent severely depressed LV function necessitating left ventricular assist device implantation or heart transplantation.

Echocardiographic parameters associated with impaired improvement have been identified in the IPAC study [14]. An initial LVEF <30% and a left ventricular end-diastolic diameter >60 mm are independent predictors of worse outcome. MRI-based data suggest right ventricular dysfunction as a predictor of worse outcome [38]. These patients may particularly benefit from bromocriptine treatment [Haghikia et al., submitted data].

## Contraception and subsequent pregnancies

Women with heart failure (independent of the etiology) are prone to further deterioration during (subsequent) pregnancy. Most heart failure drugs are contraindicated during pregnancy and lactation. Hence, safe and consistent contraception is strongly recommended in these women to avoid adverse events and prevent teratogenicity [4]. Simple barrier methods are not recommended because of their unreliability. Due to potential adverse effects, estrogen-containing methods are also not recommended in heart failure patients. Instead, copper and levonorgestrel-releasing intrauterine devices, and progesterone-only oral contraception are safe and do not harm heart failure patients. After completion of family planning and for patients with persistent severe heart failure (NYHA class III/IV, LVEF <30%), tubal ligation might be a safe option.

**Fig. 2:**
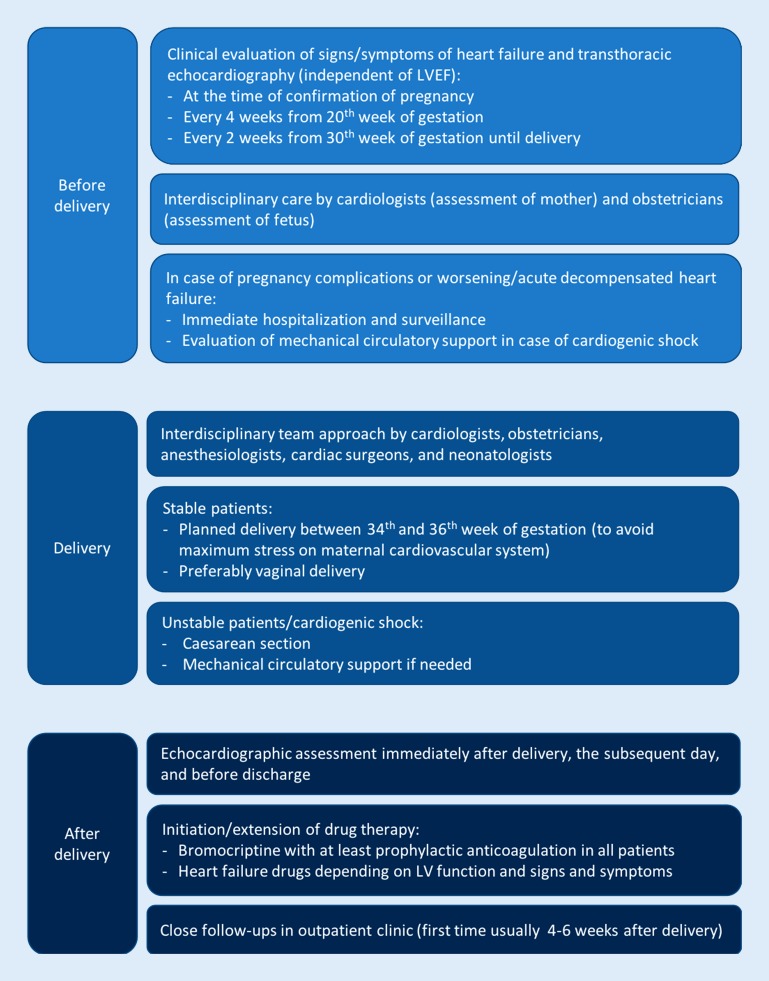
Management scheme for women with subsequent pregnancies after peripartum cardiomyopathy at Hannover Medical School. *LVEF* left ventricular ejection fraction

Women entering a subsequent pregnancy (SSP) with a persistent LV dysfunction are at a significantly higher risk for heart failure complications compared with those with recovered LV function (more heart failure symptoms, lower LVEF, and higher maternal mortality) [39]. Although SSP in women with full recovery is associated with better outcome, essentially all PPCM patients have a risk of relapse, heart failure, and death [40]. The role of bromocriptine treatment in SSP was unclear until recently. A recent study analyzed SSP in PPCM patients from Scotland, South Africa, and Germany [20]. The addition of bromocriptine to standard heart failure therapy immediately after delivery was associated with a favorable outcome. LVEF before entering the SSP did not differ between the groups. Women treated with bromocriptine had a significantly higher LVEF compared with those not receiving bromocriptine. Furthermore, the overall full recovery rate was significantly higher in the bromocriptine group. Therefore, bromocriptine is also recommended in PPCM patients with an SSP independent of LV function [40]. The management scheme of the Hannover Medical School for women with an SSP after PPCM is depicted in Fig. 2.

## Conclusion

PPCM is a rare and potentially life-threatening heart disease with a significant maternal morbidity and mortality rate. Early diagnosis based on transthoracic echocardiography and natriuretic peptides is essential. Individualized therapies by experienced physicians are necessary to optimize maternal outcome. Bromocriptine improves outcome and should be added to standard heart failure therapy. The BOARD regime represents an efficient disease-specific treatment concept for patients with acute PPCM.
